# Exploratory Analysis of the Neutrophil-to-Lymphocyte Ratio (NLR) and Mucositis Severity in Head and Neck Cancer Patients Undergoing Radiotherapy-Based Treatment: A Retrospective Study

**DOI:** 10.3390/jcm15103866

**Published:** 2026-05-18

**Authors:** Bianca Santo, Matteo Romanello, Paola De Franco, Elisa Cavalera, Donatella Russo, Giulia Lezzi, Dino Rubini, Antonio Palumbo, Giuseppe Rubini, Angela Sardaro

**Affiliations:** 1 Radiotherapy, “Vito Fazzi” Hospital, 73100 Lecce, Italy; 2 Otorhinolaryngology, Head and Neck Surgery, “Vito Fazzi” Hospital, 73100 Lecce, Italy; 3 UOC Radiotherapy, Istituto Oncologico Veneto, 35128 Padova, Italy; 4 Radiation Therapy Unit, Department of Precision Medicine, Università Degli Studi Della Campania Luigi Vanvitelli, 80129 Napoli, Italy; 5 Nuclear Medicine Unit, Interdisciplinary Department of Medicine, University of Bari, 70124 Bari, Italy

**Keywords:** head and neck cancer, neutrophil-to-lymphocyte ratio, oral mucositis, MDADI, OMAS, CTCAE

## Abstract

**Background/Objectives:** The neutrophil-to-lymphocyte ratio (NLR) is a simple biomarker reflecting systemic inflammatory status and has been investigated in head and neck cancer (HNC) as a potential prognostic indicator. Its role in relation to radiotherapy-related toxicity remains uncertain. The aim of this study was to provide a descriptive evaluation of NLR values in relation to oral mucositis severity and swallowing-related quality of life in patients undergoing radiotherapy-based treatment. **Methods:** We retrospectively evaluated 32 patients with locally advanced HNC treated with radiotherapy, with or without concomitant chemotherapy, in the definitive or adjuvant setting (March 2025–January 2026). NLR was calculated at baseline (T0), at a predefined mid-treatment timepoint (T3), and during week 6 of treatment (T6). Mucositis severity was assessed using CTCAE and the Oral Mucositis Assessment Scale (OMAS), while swallowing-related quality of life was measured using the MD Anderson Dysphagia Inventory (MDADI). Relationships between NLR values and toxicity endpoints were descriptively assessed using Spearman correlation analysis. **Results:** No statistically significant correlations were observed between NLR values and OM severity or swallowing-related outcomes at any evaluated timepoint. At T3, non-significant correlations were observed between NLR and CTCAE mucositis grade and between NLR and MDADI global score. No statistically significant correlations were observed between NLR values and OMAS at any evaluated timepoint. **Conclusions:** In this retrospective cohort, no association between NLR and radiotherapy-related mucositis severity or swallowing-related quality of life was demonstrated. These findings are descriptive and limited by the small sample size, the retrospective design, and the absence of control for potential confounding factors. No inferential or causal conclusions can be drawn. Further prospective studies with larger and more homogeneous cohorts are required to better characterize NLR behavior in this clinical setting.

## 1. Introduction

Head and neck cancer (HNC) comprises a heterogeneous group of malignancies for which radiotherapy (RT) represents a cornerstone of treatment, either as a definitive approach or as an adjuvant strategy following surgical resection. In patients with locally advanced disease, radiotherapy is frequently combined with concurrent chemotherapy to improve locoregional control and survival outcomes. Despite its therapeutic effectiveness, radiotherapy-based treatment is associated with a substantial risk of acute toxicities, among which oral mucositis (OM) is one of the most common, debilitating, and dose-limiting complications [1,2,3].

Oral mucositis affects the majority of patients undergoing radiotherapy for HNC and may present with a wide spectrum of clinical manifestations, ranging from mild erythema to severe ulceration requiring opioid analgesia, nutritional support, or treatment interruption [2,4]. Beyond local symptoms, OM has a significant impact on quality of life, particularly in relation to pain, swallowing difficulties, and nutritional status, and may contribute to reduced treatment adherence and potentially compromised oncological outcomes [5,6]. The pathobiology of OM is complex and involves a sequence of overlapping phases, including initial tissue injury, inflammatory amplification, ulceration, and healing [7]. These processes are driven by interactions between epithelial cells, submucosal tissues, inflammatory mediators, and the oral microbiome. During the ulcerative phase, disruption of the mucosal barrier facilitates microbial colonization, further amplifying inflammatory responses and tissue damage [7,8]. While treatment-related factors such as radiation dose, irradiated volume, and concomitant chemotherapy are recognized determinants of mucositis severity, inter-patient variability suggests that host-related factors may also play a role [9]. Systemic inflammation has been implicated in both cancer progression and treatment-related toxicity. Biomarkers reflecting inflammatory and immune status may therefore provide additional insight into individual variability in treatment tolerance. The neutrophil-to-lymphocyte ratio (NLR) is an easily obtainable and inexpensive parameter derived from routine blood tests, which has been widely investigated as a prognostic marker in several solid tumors, including HNC. Elevated baseline NLR values have been associated with adverse oncological outcomes, including reduced survival and increased tumor aggressiveness [10,11,12]. In addition to its prognostic role, interest in NLR as a parameter reflecting treatment-related systemic inflammatory changes has increased. In the context of radiotherapy, fluctuations in inflammatory markers during treatment may occur in parallel with tissue injury and repair processes. While the prognostic role of NLR in head and neck cancer has been widely investigated, its potential relevance in relation to radiotherapy-related acute toxicity remains less clearly defined [13,14]. In particular, evidence regarding NLR values during radiotherapy and their relationship with oral mucositis severity or swallowing-related quality of life is limited and heterogeneous. Most available studies have focused on baseline NLR or selected treatment settings, whereas data describing NLR at multiple predefined timepoints during radiotherapy remain scarce [15,16,17]. Therefore, the present study was designed as a descriptive analysis of NLR values at multiple predefined timepoints during radiotherapy-based treatment in patients with head and neck cancer. Specifically, we described NLR values in relation to oral mucositis severity and swallowing-related quality of life, assessed using validated clinician-reported, objective, and patient-reported instruments. No inferential or causal assumptions were made, and the analysis was intended to provide a descriptive overview within the limitations of a retrospective real-world dataset.

## 2. Materials and Methods

Patients with locally advanced HNC treated with definitive or adjuvant radiotherapy-based strategies between March 2025 and January 2026 were retrospectively evaluated. This was a single-institution, retrospective observational cohort study. Patients were eligible if they had histologically confirmed locally advanced head and neck cancer and were treated with radiotherapy, either alone or in combination with chemotherapy, in the definitive or adjuvant setting. Inclusion required the availability of complete clinical data, full blood count values for NLR calculation at predefined timepoints, and toxicity assessments including CTCAE, OMAS, and MDADI.

Patients were excluded in case of incomplete laboratory or toxicity data at the selected timepoints. Due to the retrospective nature of the study, clinical variables potentially influencing systemic inflammatory status—such as infections, corticosteroid use, and other acute inflammatory conditions during treatment—were not systematically recorded and were therefore not included as exclusion criteria or covariates. This represents a potential source of unmeasured confounding.

For each patient, NLR was calculated at baseline (T0), at a predefined mid-treatment assessment timepoint (T3), and during the sixth week of treatment (T6). T3 was selected as a standardized assessment point during treatment; individual variability in toxicity onset and severity was not accounted for. Mucositis severity was assessed using the Common Terminology Criteria for Adverse Events (CTCAE) and the Oral Mucositis Assessment Scale (OMAS), while swallowing-related quality of life was evaluated using the MD Anderson Dysphagia Inventory (MDADI). Clinical evaluations were performed weekly during radiotherapy, and at T0, T3, and T6, patients underwent dedicated otolaryngological assessment for OMAS scoring and MDADI administration. Correlations between NLR and toxicity endpoints (CTCAE, OMAS, and MDADI) were assessed using Spearman’s rank correlation coefficient at each predefined timepoint. Statistical significance was defined as *p* < 0.05. The analysis was exploratory and descriptive in nature. Given the limited sample size, multivariable models and longitudinal analyses were not performed due to the risk of overfitting and unstable estimates. No correction for multiple comparisons was applied; therefore, the results should be interpreted with caution, as the risk of type I error cannot be excluded. Furthermore, the study design does not allow adjustment for potential confounding factors, including treatment modality, tumor subsite, chemotherapy exposure, or unrecorded inflammatory conditions, which may influence NLR values. This study was conducted in accordance with the principles of the Declaration of Helsinki and applicable national regulations governing retrospective observational research. The analysis was based exclusively on anonymized clinical data collected during routine care, without any intervention beyond standard clinical management. All patients had provided written informed consent for the use of their clinical data for research purposes. According to institutional practice and the regulatory framework applicable to non-interventional retrospective studies using anonymized data, formal ethics committee approval was not required.

## 3. Results

A cohort of 32 patients with head and neck cancer treated with radiotherapy-based strategies was retrospectively analyzed. Patient characteristics are summarized in Table 1. Treatment approaches included definitive radiotherapy (RT) or concurrent chemoradiotherapy (CRT), as well as adjuvant RT or adjuvant CRT following surgical resection, according to tumor stage, pathological features, and clinical indication. NLR values were available for all included patients at baseline (T0), at a predefined mid-treatment timepoint (T3), and during the sixth week of treatment (T6). Clinical mucositis severity and functional outcomes were evaluated at corresponding timepoints.

At baseline (T0), no statistically significant correlation was observed between NLR and oral mucositis severity as assessed by the Common Terminology Criteria for Adverse Events (CTCAE) (Spearman’s ρ = −0.32; *p* = 0.095). Similarly, no statistically significant correlations were observed between NLR and oral mucositis assessed using the Oral Mucositis Assessment Scale (OMAS), nor with swallowing-related quality of life as measured by the MD Anderson Dysphagia Inventory (MDADI) Global Score. At the mid-treatment timepoint (T3), no statistically significant correlations were identified between NLR and any of the evaluated endpoints. A non-significant positive correlation was observed between NLR and CTCAE mucositis grade (Spearman’s ρ = 0.26; *p* = 0.166). No statistically significant correlation was observed between NLR and OMAS score at T3. A non-significant inverse correlation was observed between NLR and MDADI Global Score (Spearman’s ρ = −0.18; *p* = 0.340).

During the sixth week of treatment (T6), no statistically significant correlations were identified between NLR and the evaluated clinical or functional endpoints. NLR was not significantly associated with CTCAE mucositis grade, OMAS score, or MDADI Global Score (Spearman’s ρ = −0.30; *p* = 0.106).

**Figure 1:**
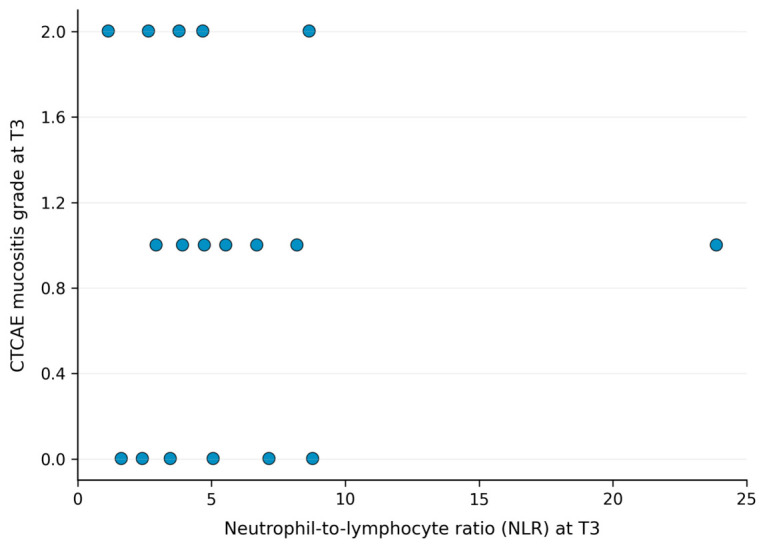
Scatter plot showing the relationship between neutrophil-to-lymphocyte ratio (NLR) and CTCAE mucositis grade at the predefined mid-treatment timepoint (T3). Each dot represents an individual patient. Spearman correlation analysis showed no statistically significant correlation.

**Figure 2:**
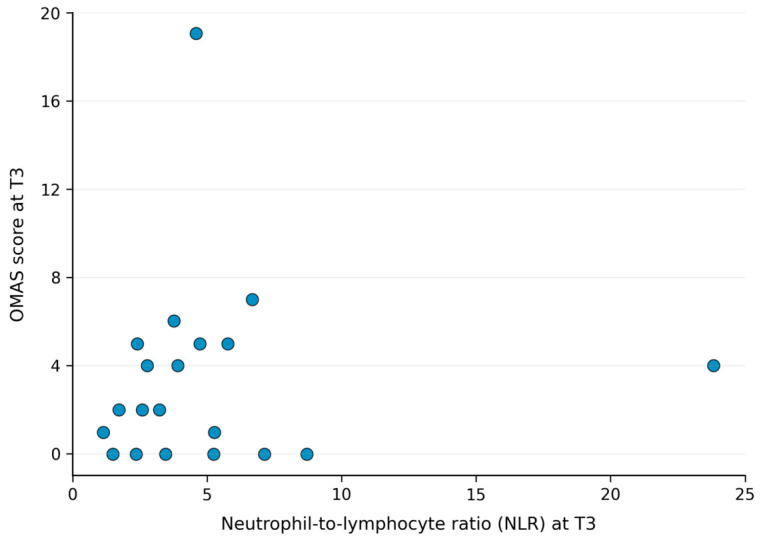
Scatter plot showing the relationship between neutrophil-to-lymphocyte ratio (NLR) and OMAS score at the predefined mid-treatment timepoint (T3). Each dot represents an individual patient. Spearman correlation analysis showed no statistically significant correlation.

**Figure 3:**
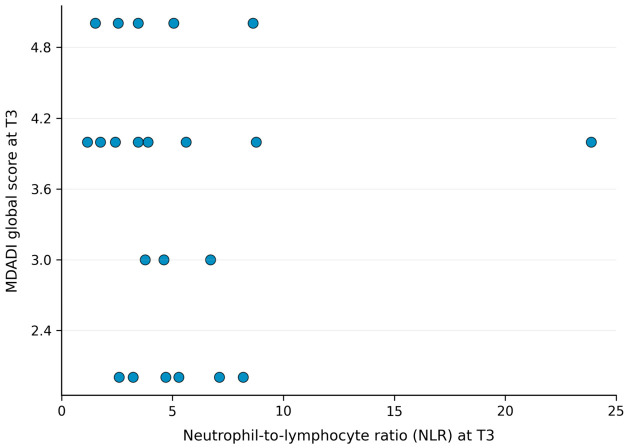
Scatter plot showing the relationship between neutrophil-to-lymphocyte ratio (NLR) and MDADI global score at the predefined mid-treatment timepoint (T3). Each dot represents an individual patient. Spearman correlation analysis showed no statistically significant correlation.

Overall, no statistically significant correlations between NLR and mucositis severity or swallowing-related outcomes were observed at any evaluated timepoint. Figure 1, Figure 2 and Figure 3 show the observed trends in the scatter plots.

## 4. Discussion

The present retrospective analysis descriptively assessed NLR values in relation to radiotherapy-related mucositis severity and swallowing-related quality of life in patients with head and neck cancer. No statistically significant correlations were observed at any evaluated timepoint. Previous studies have described possible relationships between NLR and severe mucositis [15,16,17,18], but differences in study design, patient populations, biomarker timing, and toxicity assessment limit direct comparison. In the present analysis, no statistically significant associations were observed at any evaluated timepoint, and therefore, the current findings do not support a relationship between NLR and treatment-related mucosal toxicity or functional outcomes. Several limitations should be acknowledged when interpreting these findings. The relatively small sample size limits statistical power and reduces the ability to detect moderate associations. The retrospective design precluded adjustment for relevant confounding variables, including infections, corticosteroid exposure, and other inflammatory conditions that were not systematically recorded and may have influenced NLR values. In addition, the cohort was clinically heterogeneous with respect to tumor subsite, treatment setting, and concomitant chemotherapy exposure, which further limits interpretability. From a statistical perspective, only exploratory univariate analyses were performed, without multivariable adjustment or correction for multiple comparisons. CTCAE, OMAS, and MDADI capture complementary but distinct dimensions of treatment-related toxicity, including clinician-reported grading, objective mucosal assessment, and patient-reported swallowing-related quality of life [19,20,21,22], Despite these limitations, the present study includes some methodological strengths. NLR was assessed at multiple predefined timepoints during treatment, allowing a longitudinal description of systemic inflammatory status. In addition, the combined use of clinician-reported (CTCAE), objective (OMAS), and patient-reported (MDADI) measures provides a multidimensional assessment of treatment-related toxicit [23,24,25]. Given the absence of statistically significant findings and the methodological limitations described above, the present observations should be interpreted strictly descriptively. No inferential or causal conclusions can be drawn. Overall, this study does not demonstrate an association between NLR and radiotherapy-related mucositis severity or swallowing-related outcomes in patients with HNC. Further prospective studies with larger sample sizes, standardized data collection, and appropriate control of confounding factors are required to better characterize NLR patterns in this context.

## 5. Conclusions

In this retrospective cohort of patients with head and neck cancer undergoing radiotherapy-based treatment, no association between the neutrophil-to-lymphocyte ratio (NLR) and radiotherapy-related mucositis severity or swallowing-related quality of life was demonstrated. The findings of this study are descriptive and are limited by the small sample size, the retrospective design, the clinical heterogeneity of the cohort, and the lack of systematic control for potential confounding factors influencing systemic inflammatory status. Given these limitations, no inferential or causal conclusions can be drawn regarding NLR values in the context of treatment-related toxicity. Further prospective studies with larger and more homogeneous cohorts are required to better characterize NLR patterns in this clinical setting.

## Figures and Tables

**Table 1:** Clinical and Treatment Characteristics. Study population (*n* = 32).

Variable	Category	*n* (%)
Tumor subsite		
	Oral cavity	10 (31.2)
	Larynx	9 (28.1)
	Oropharynx	6 (18.7)
	Parotid gland	2 (6.3)
	Submandibular gland	2 (6.3)
	Hypopharynx	2 (6.3)
	Maxillary sinus	1 (3.1)
Stage		
	III	20 (62.5)
	IVA	9 (28.1)
	IVB	3 (9.4)
Histology		
	Squamous cell carcinoma	25 (78.1)
	Adenocarcinoma	5 (15.6)
	Other	2 (6.3)
Grading		
	G3	20 (62.5)
	G2	11 (34.4)
	G1	1 (3.1)
Treatment setting		
	Adjuvant CRT	10 (31.2)
	Definitive CRT	10 (31.2)
	Adjuvant RT	8 (25.0)
	Definitive RT	4 (12.5)
Concomitant chemotherapy		
	Yes	21 (65.6)
	No	11 (34.4)
Metastatic disease at baseline		
	No	32 (100.0)

## Data Availability

The original contributions presented in this study are included in the article. Further inquiries can be directed to the corresponding author.
